# Circulating Conventional and Plasmacytoid Dendritic Cell Subsets Display Distinct Kinetics during *In Vivo* Repeated Allergen Skin Challenges in Atopic Subjects

**DOI:** 10.1155/2014/231036

**Published:** 2014-04-28

**Authors:** Stelios Vittorakis, Konstantinos Samitas, Sofia Tousa, Eleftherios Zervas, Maria Aggelakopoulou, Maria Semitekolou, Vily Panoutsakopoulou, Georgina Xanthou, Mina Gaga

**Affiliations:** ^1^7th Respiratory Department and Asthma Centre, Athens Chest Hospital, Athens 11527, Greece; ^2^Cellular Immunology Laboratory, Division of Cell Biology, Center for Basic Research, Foundation for Biomedical Research of the Academy of Athens, Athens 11527, Greece

## Abstract

Upon allergen challenge, DC subsets are recruited to target sites under the influence of chemotactic agents; however, details pertinent to their trafficking remain largely unknown. We investigated the kinetic profiles of blood and skin-infiltrating DC subsets in twelve atopic subjects receiving six weekly intradermal allergen and diluent injections. The role of activin-A, a cytokine induced in allergic and tissue repair processes, on the chemotactic profiles of DC subsets was also examined. Plasmacytoid (pDCs) and conventional DCs (cDCs) were evaluated at various time-points in the blood and skin. *In situ* activin-A expression was assessed in the skin and its effects on chemokine receptor expression of isolated cDCs were investigated. Blood pDCs were reduced 1 h after challenge, while cDCs decreased gradually within 24 h. Skin cDCs increased significantly 24 h after the first challenge, inversely correlating with blood cDCs. Activin-A in the skin increased 24 h after the first allergen challenge and correlated with infiltrating cDCs. Activin-A increased the CCR10/CCR4 expression ratio in cultured human cDCs. DC subsets demonstrate distinct kinetic profiles in the blood and skin especially during acute allergic inflammation, pointing to disparate roles depending on each phase of the inflammatory response. The effects of activin-A on modulating the chemotactic profile of cDCs suggest it may be a plausible therapeutic target for allergic diseases.

## 1. Introduction

Allergic diseases, such as asthma and atopic dermatitis, are on the rise in western societies and pose a significant burden for patients and health care systems. Allergic inflammation relates to excessive T helper type 2 (T_H2_) cell-mediated responses against innocuous environmental allergens. Dendritic cells (DCs) are known sentinels of the immune system entrusted with the chief task of antigen recognition, presentation, and T cell activation. Different DC subsets with diverse functionalities have been described, depending on the level of expression of specific surface markers, their activation status, and anatomic location. There are two major human DC subsets: myeloid or conventional DCs (cDCs), generally considered immunogenic [1, 2], and plasmacytoid DCs (pDCs), which can exhibit suppressive functions on allergen-driven T_H2_ cell-mediated responses [3]. Pertinent to the skin, cDCs constitute the major resident DC population in normal human dermis and are characterized by CD1c expression (also known as blood dendritic cell antigen-1 (BDCA)-1) [4, 5]. Plasmacytoid DCs are present in the skin and can be readily identified by expression of CD303, also known as BDCA-2 [6, 7].

Increased numbers of DC subsets have been previously reported in the blood, nasal, and/or lung mucosa of subjects with atopy, allergic rhinitis, and/or asthma [8–11], suggesting that specific DC subgroups are induced in response to allergic inflammation. Nevertheless, few clinical studies have addressed DC kinetics in allergic responses, and these involve mostly allergen inhalation or segmental bronchial allergen challenge [12–17]. Understanding of the trafficking of human DCs upon allergen challenge* in vivo* is essential for controlling the balance between immunity and tolerance.

DC migration is guided by the rapid upregulation of adhesion molecules and chemokine receptors in response to chemokine and cytokine gradients generated by tissue-resident and infiltrating immune cells. Activin-A is a pleiotropic cytokine-member of the TGF-*β* superfamily of proteins. It is produced by inflammatory and structural tissue cells and acts as an important regulator of allergic inflammation [18–20] and skin repair processes [21]. In fact, recent studies have revealed potent anti-inflammatory effects of activin-A* in vivo* during allergen-induced cutaneous sensitization [22]. Activin-A is produced by different DC subsets, promotes DC differentiation, and affects the ability of mature DCs to take up antigens [23]. In addition, activin-A is involved in the differentiation and migration of human Langerhans dendritic cells, mostly through the regulation of chemokines and chemokine receptor networks [24, 25]. Still, the effects of activin-A on shaping the chemotactic responses of human DC subsets during exposure to allergen* in vitro* remain elusive.

In the present study, we hypothesized that circulating cDCs and pDCs exhibit different kinetic profiles* in vivo* upon repeated skin allergen challenges, reflecting their distinct roles during acute and established chronic inflammation. To explore this, we used a well-established human* in vivo* model of repeated skin allergen challenges. Furthermore, we examined activin-A expression in the inflamed skin and explored possible correlations with DC subset infiltration following allergen challenge. Finally, the effects of activin-A on the chemokine receptor profile of allergen stimulated human CD1c^+^ DCs were also investigated.

## 2. Methods

### 2.1. Study Population and Design

Healthy nonsmoking volunteers between the ages of 18 and 55 were initially screened and subjected to skin prick testing (SPT) to a panel of 18 common aeroallergens (HAL Allergy, Benelux). Total blood counts, serum biochemistry, total IgE levels measurements, and spirometry were performed. A total of twelve subjects with strong positive SPT reaction to* Dermatophagoides pteronyssinus*, the European house dust mite, were enrolled in the study. Subject characteristics are summarized in Table 1. All subjects signed an informed consent form before enrolment. The study was approved by the “Sotiria” Hospital Research Ethics Committee and the Greek National Organization for Medicines and was conducted according to the Declaration of Helsinki principles.

**Figure 1:**
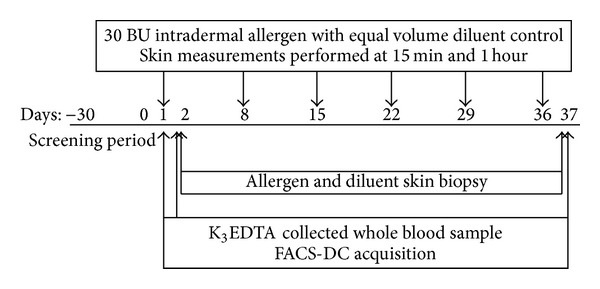
Study design. Flow diagram showing the time-points when the skin challenges with allergen and diluent were performed and when the samples were taken. The dose of* Dermatophagoides pteronyssinus* administered was 30 BU at the allergen site with equal volume of diluent at the opposing site every week (solid arrows). A screening period of 2 weeks was introduced to clinically verify that subjects did not exhibit seasonal allergic symptoms or an upper/lower respiratory infection.

All participants were free of atopic symptoms for at least one month before and during the study. Participants had no history of infection and had not received any treatment with oral/inhaled corticosteroids, antihistamines, anti-IgE, or antileukotrienes for one month before and during the study. Each subject received a total of six weekly intradermal injections of allergen, to which they were sensitive, and diluent on the extensor aspect of the left and right forearms, respectively. Skin biopsies were obtained 24 h after the first challenge and 24 h after the last challenge at both allergen and diluent sites. Whole blood was taken before the first challenge, 1 h after the first challenge, and just before each biopsy acquisition. The study design is depicted in Figure 1.

### 2.2. *In Vivo* Allergen Challenges

A 21-gauge needle was used to deliver 100 *μ*L (30 BU) of allergen aqueous solution (*Dermatophagoides pteronyssinus*, Allergopharma Joachim Ganzer KG, Germany) intradermally at the same site on the extensor aspect of the left forearm. The same diluent volume (0.9% sodium chloride) was administered in the same manner on the extensor aspect of the right forearm. In this way, each patient served as his/her own control. The challenge tests were performed at the same time each day. Measurements of skin reactions were performed at 15 min, 1 h, and 6 h, as previously described [26], by a single investigator throughout the study.

### 2.3. Preparation of Skin Biopsies

Skin specimens were obtained from the centre of both allergen and diluent sites in each subject using a 4 mm punch biopsy tool (Stiefel Laboratories). Local anaesthesia was induced by injecting subcutaneously 0.5 mL of 2% lidocaine hydrochloride. Tissue samples from the same arm after the last challenge were taken 0.8 cm apart. After appropriate orientation and handling, specimens were cut in half and one of the resulting tissue samples was embedded in OCT medium and snap-frozen in isopentane (BDH Chemicals) precooled in liquid nitrogen, while the other was fixed in formalin overnight and embedded in paraffin. Frozen specimens were stored at 80°C.

### 2.4. Immunohistochemistry

Paraffin sections 4-5 mm thick were deparaffinised in xylol, rehydrated in graded alcohol series, and immunostained for activin-A, as previously described [19]. In brief, the alkaline phosphatase-antialkaline phosphatase method was used, and specific antibody binding was visualized using Vectastain ABC-AP kits and the Fast-Red chromogen (rabbit anti-goat IgG, AK-5005 Vector Laboratories). All incubations were performed at room temperature. Washes were performed in PBS. Normal rabbit serum (10%) was used to reduce nonspecific binding. An affinity-purified polyclonal goat antibody against human activin-A and a goat IgG control antibody (which served as negative control) were used as primary antibodies (AF338 and AB-108-C, resp., R&D Systems).

### 2.5. Immunofluorescence Staining

To determine the numbers of cDCs and pDCs in the skin, we applied an immunostaining technique to serial cryosections (6 *μ*m). Slides were warmed up at room temperature (RT) for 30 min, fixed in ice cold (4°C) acetone for 5 min, air dried for 45 min, and washed in PBS. Sections were then blocked with normal goat serum (S-1000, Vector Laboratories) and incubated overnight at 4°C with unconjugated mouse anti-human CD1c (BDCA-1) for the determination of cDCs or mouse anti-human CD303 (BDCA-2) for pDCs (130-090-695 and 130-090-690, resp., Miltenyi Biotec). A secondary goat anti-mouse antibody conjugated with AlexaFluor568 or goat anti-mouse conjugated with AlexaFluor488 (A21134 and A21121, resp., Molecular Probes) was applied for 30 min at RT. Negative controls were obtained by substitution of the primary antibody with the same concentration of the corresponding IgG isotype control (MAB003 and MAB002, resp., R&D Systems). Slides were rinsed and then counterstained with Hoechst (H3569, Molecular Probes).

### 2.6. Confocal Laser Microscopy

Skin sections processed with immunofluorescence staining were examined using a confocal laser microscope. Fluorescent images of the tissue sections were acquired, using a Leica DMI6000 inverted microscope with DIC optics (Leica TCS SP5). Hoechst was excited by the 405 nm laser diode and the fluorescence was collected using a long-pass (LP) 420 emission filter; the green (AF488) antibody was excited by the 488 nm argon ion laser line and the fluorescence was collected using a band-pass (BP) 505–530 emission filter; the red (AF568) antibody was excited by the 568 nm green helium-neon laser line and the fluorescence was collected using an LP560 emission filter. Tissue sections were visualised using the confocal microscope at 1,024 × 1,024-pixel resolution through a HCX PL APO CS 20.0 × 0.70 DRY UV with eight-times averaging in sequential scanning (multitrack) mode with the pinhole set to obtain an optical section of approximately 1 mm in all channels.

### 2.7. Quantification of DCs and Activin-A Positive Cells

Images of tissue sections were recorded using a computerized image analysis system (AxioVision, Carl Zeiss). Cells stained for activin-A were counted in the epidermis and dermis by using an AxioScopeA1 light microscope (Carl Zeiss). Results were normalized to the area of the epithelium and to the length of the basement membrane and expressed as number of cells per mm^2^. Quantitative measurements of BDCA-1 and BDCA-2 positive cells in skin tissue specimens were performed as previously described [5, 7]. DCs that showed positive staining were counted to a dermis depth of 500 *μ*m. Stained DCs were regarded positive when showing DC morphology and were expressed as number of cells per mm^2^ of dermis area. Activin-A, BDCA-1, and BDCA-2 staining was performed in serial tissue sections, which were coded and examined in a blinded manner at the end of the study by two investigators. The intra- and interobserver variations were calculated to be less than 4% and 8%, respectively.

### 2.8. CD1c^+^ DC Isolation and* In-Vitro* Stimulation

Peripheral blood mononuclear cells (PBMCs) were obtained by Ficoll gradient centrifugation and CD1c^+^ DCs were isolated using the CD1c^+^ (BDCA-1^+^) human dendritic cell isolation kit, (Miltenyi Biotec 130-090-506). Briefly, in a first step, PBMCs were magnetically labeled with CD19 microbeads. Using this approach, CD19^+^ cells were depleted by separation over a MACS column which was placed in the magnetic field of a MACS separator. In a second step, CD1c^+^ DCs, in the B cell-depleted flow-through fraction, were magnetically labeled with CD1c-biotin and antibiotin microbeads. Upon separation, the labeled CD1c^+^ DCs were retained within the column and eluted after removing the column from the magnetic field. This two-step procedure allows the isolation of purified CD1c^+^ DCs (with a purity >80%). A total of 5 × 10^4^ CD1c^+^ DCs were placed in 96 flat-bottom plates and cultured for 24 h with (a) medium, (b) 100 ng/mL LPS, or (c) 1 *μ*g/mL* Dermatophagoides pteronyssinus* in the presence or absence of 50 ng/mL recombinant activin-A (R&D). LPS is a TLR4 ligand extensively used for the activation of DCs and served as a positive control [27]. Following 24 h of culture, cells were stained with fluorochrome-conjugated antibodies against human CCR4 (CD194), CCR6 (CD196), CCR9 (CD199), CXCR3 (CD183), and CCR10 (all from Biolegend) and analyzed by flow cytometry.

### 2.9. Flow-Cytometric Analysis

**Figure 2:**
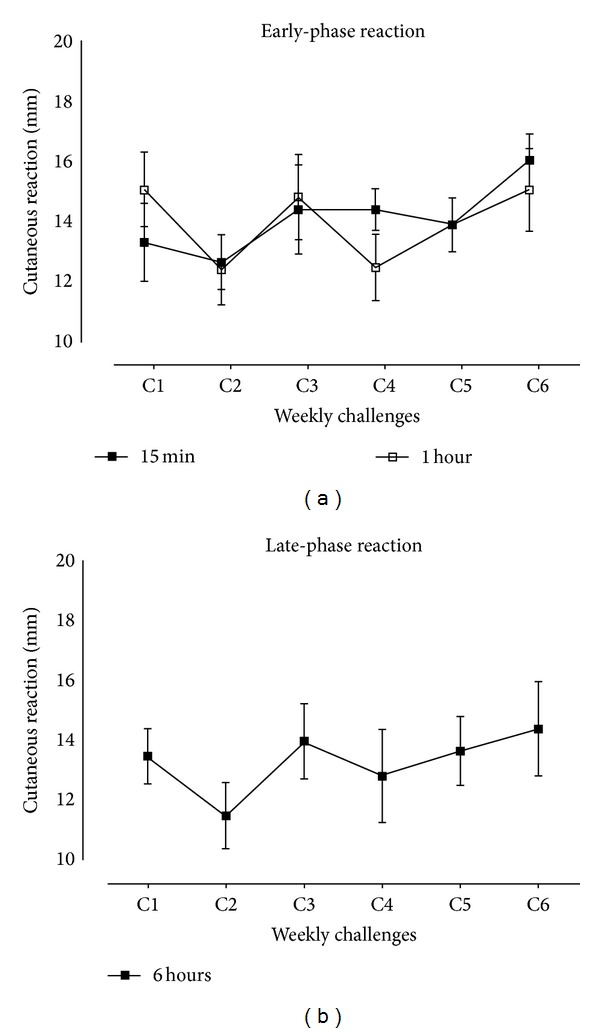
Early- and late-phase reactions. Mean diameter of skin reactions after each allergen challenge with* Dermatophagoides pteronyssinus* measured at 15 min and 1 h (a), as well as 6 h (b), is presented in mm. No statistical significant differences were observed between challenges or at different time-points. Values are expressed as mm ± SEM.

Peripheral blood was drawn and processed for flow-cytometric analysis within 30 min of collection. The identification of peripheral DC subsets was based on two different three-color assays (IOTest PN-A07405 “myeloid subset” and PN-A07412 “plasmacytoid subset,” Immunotech, Marseille, France) and was performed as previously described [28]. Whole blood (100 *μ*L) was mixed with 20 *μ*L of each monoclonal antibody or appropriate labelled isotype control cocktail, vortexed for 1 sec, and incubated for 30 min at RT, protected from light. The samples were lysed and fixed (VersaLyse PN-IM3648 and fixative solution PN-IM3515, Beckman Coulter, Krefeld, Germany), washed, resuspended in PBS, and kept on ice until flow cytometric analysis. At least 300000 events were acquired on a dual-laser Beckman-Coulter FC500. Further analysis of the flow-cytometric raw data was performed with the FLOWJO software (Tree Star Inc., USA). The gating strategy used for the analysis of DC subsets is provided in Figure 2.

### 2.10. Statistical Analysis

Data are expressed as median with interquartile range (first and third quartiles) unless specified otherwise. Normality was assessed using D' Agostino—Pearson omnibus normality tests. Data differed from normal distribution and were, thus, analyzed with non-parametric statistics. Statistical analysis of skin reactions measured at 15 min, 1 h and 6 h after each challenge was performed by two-way ANOVA mixed model, followed by Bonferroni* post hoc* analysis. DC kinetics in peripheral blood as well as the analysis of the number of tissue DC subsets at different time-points were performed by repeated measures nonparametric one-way ANOVA (Friedman test), followed by Dunn's* post hoc* analysis for all pairs. The correlation between DC subsets in skin tissue and peripheral blood at different time-points, as well as between activin-A tissue expression and DC subsets in the skin, was performed by nonparametric Spearman correlation tests. Two-group analysis of the effects of activin-A's on chemokine receptor expression by allergen-stimulated CD1c^+^ DCs was performed by the Mann-Whitney *U* test. A *P* value of ≤0.05 was considered statistically significant. Data storage and analysis were performed with statistical analysis software (GraphPad Prism v5, GraphPad Inc., CA, USA).

## 3. Results

### 3.1. Early- and Late-Phase Allergen-Induced Cutaneous Reactions

The mean diameter of skin reactions after each allergen challenge measured at 15 min, 1 h, and 6 h (Figure 2). Statistical analysis showed no diameter differences between challenges or at different time-points, minimizing the possibility of induction of tolerance during the* in vivo* protocol. No indurations were observed at the diluent site. No diluent and/or allergen-related adverse events were observed in any of the subjects.

### 3.2. DC Subsets Exhibit Different Kinetic Patterns in the Peripheral Blood Following Repeated Allergen Skin Challenges* In Vivo*

**Figure 3:**
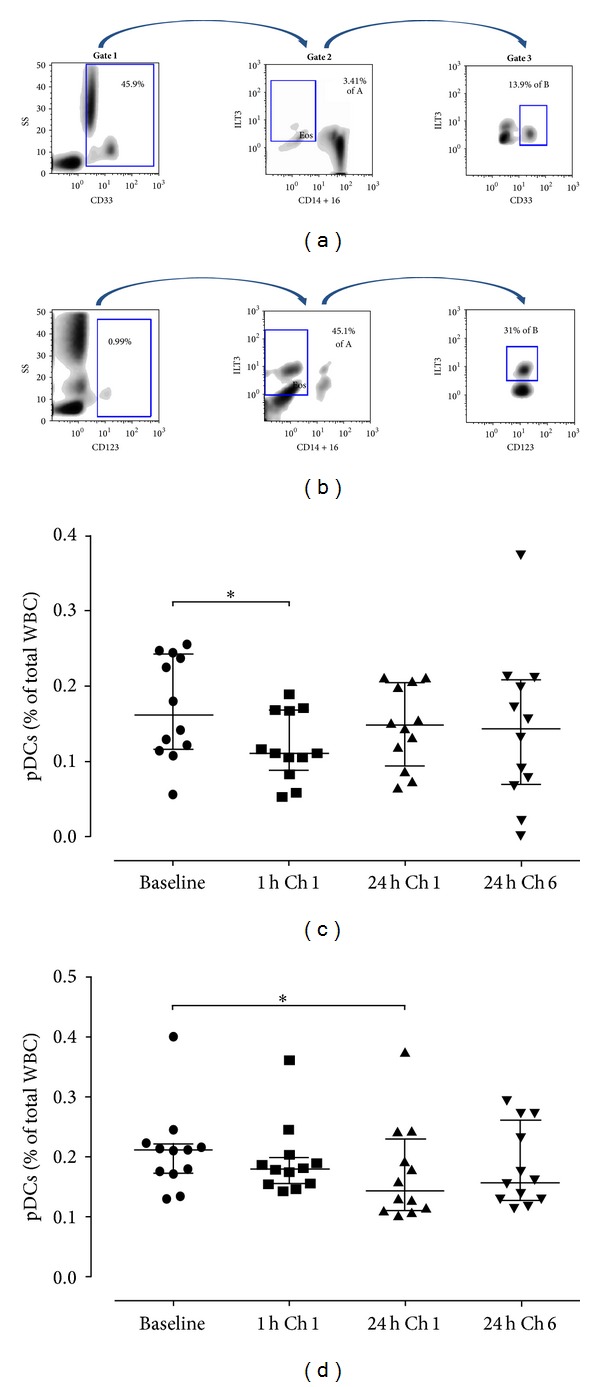
DC subsets exhibit different kinetic patterns in the peripheral blood following repeated allergen skin challenges* in vivo*. Gating strategies utilized to identify cDC and pDC subsets in the peripheral blood by flow cytometry. (a) For cDC identification, a 3-step analysis was performed. Initially, CD33^pos^ cells were selected (Gate 1) to differentiate between mature lymphoid cells or lymphoid precursors (CD33^neg^) from other cells of myeloid origin that include cDCs. Next, all CD(14+16)^dim⁡  to  neg^/ILT3^pos^ cells are selected in Gate 2 to exclude monocytes, macrophages, NK cells, and neutrophils. Gate 3 is drawn around CD33^bright^/ILT3^pos^ cells, so cDCs are characterized as CD33^bright^/ILT3^pos^/CD(14+16)^dim⁡ to neg^. (b) Regarding pDCs, initially all CD123^pos^ cells are selected (Gate 1) and then gated on the basis of CD(14+16)^neg^ expression (Gate 2) to exclude monocytes, lymphocytes, and most granulocytes. Gate 3 is drawn around CD123^bright^/ILT3^bright^ cells, strictly selecting pDCs and excluding basophils, so pDCs are characterized as CD123^bright^/ILT3^bright^/CD(14+16)^neg^. Representative FACS plots are shown. The percentages of pDCs (c) and cDCs (d) in the peripheral blood at baseline and following* in vivo* allergen challenges are shown. Data are expressed as median with interquartile range (first and third quartiles).

Circulating cDCs and pDCs were assessed before allergen challenge, 1 h and 24 h after the first allergen challenge, and 24 h after the sixth challenge (Figures 3(a) and 3(b), resp.). Interestingly, our data revealed significant differences for both cDC and pDC numbers in the peripheral blood of atopics at distinct time-points following* in vivo* allergen skin challenge, compared to baseline (*P* < 0.05, resp.).* Post hoc* analysis revealed that changes in the pDC population were very rapid, as they were significantly decreased 1 h after the first allergen challenge and returned to baseline after 24 h (Figure 3(c)). On the other hand, cDCs started to decrease by the first hour after challenge and continued to decrease, reaching significantly lower levels at 24 h, with a later-on trend to return to baseline after 5 weeks (Figure 3(d)).

### 3.3. Conventional DCs Are Recruited to the Skin Early Following* In Vivo* Allergen Challenge

**Figure 4:**
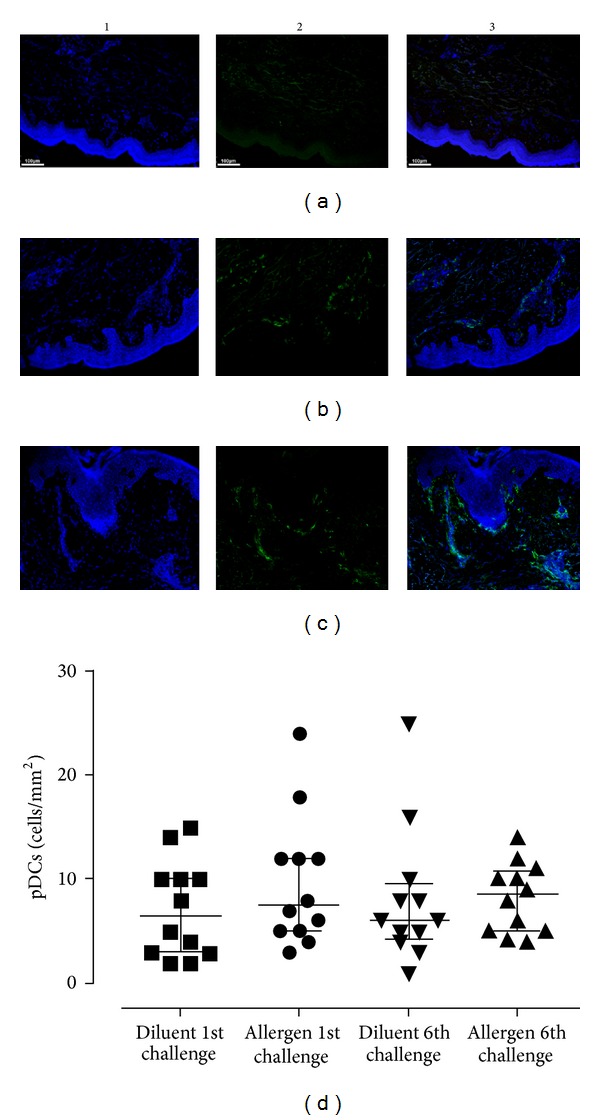
Plasmacytoid DC kinetics in the skin upon* in vivo* allergen challenge. Immunofluorescence staining was performed on skin biopsies and examined by confocal microscopy. Counterstaining was performed with Hoechst to visualize nuclear DNA (blue, column 1). PDCs were stained with a monoclonal antibody against BDCA-2 and envisioned with a secondary goat anti-mouse antibody conjugated with AF488 (green, column 2). Column 3 is the result of merging columns 1 and 2. Representative microphotographs (×100) of pDCs are shown 24 h after the first challenge at the diluent (a) and allergen sites (b) and after the sixth challenge at the allergen site (c). PDC numbers were not significantly altered between different time-points, although a trend for increased pDCs was observed after the sixth allergen challenge (d). Data are expressed as median with interquartile range (first and third quartiles). *P < 0.05, WBC: whole blood cells.

**Figure 5:**
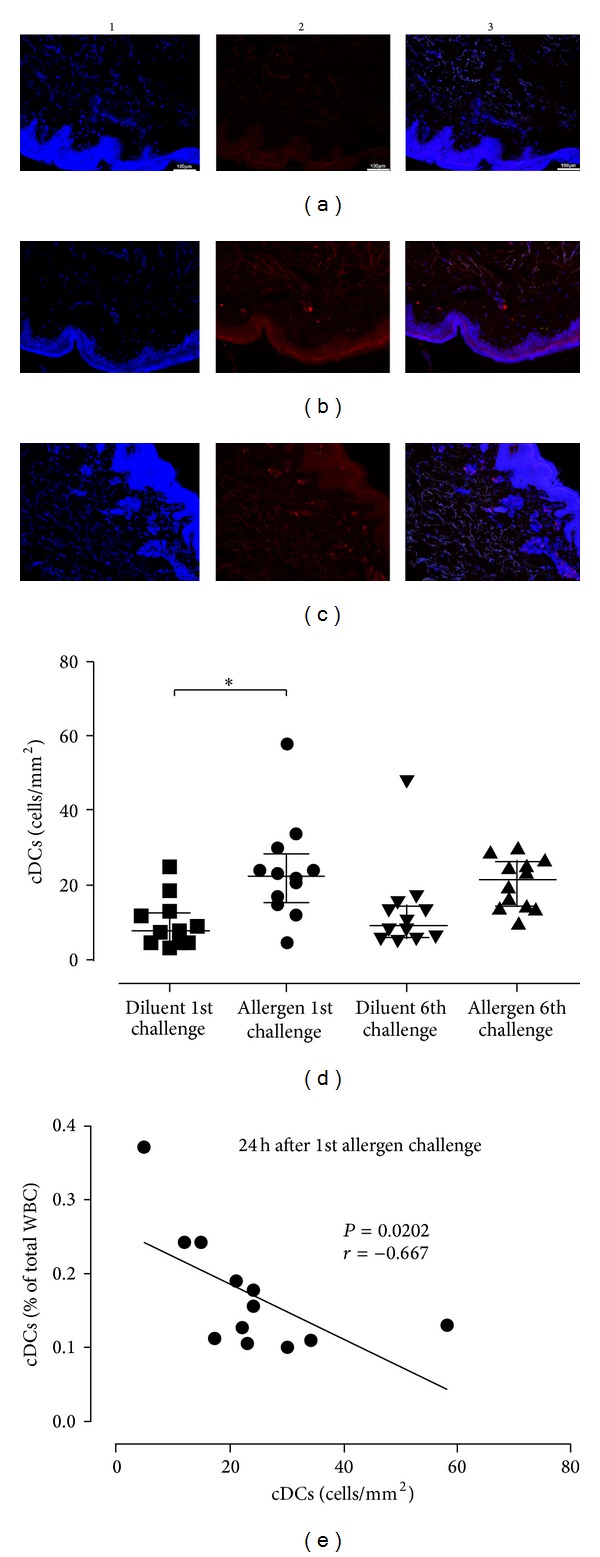
Conventional DCs are recruited early to the skin upon* in vivo* allergen challenge. Immunofluorescence staining and data analysis were performed as described in Figure 4. Counterstaining was performed with Hoechst (blue, column 1). CDCs were stained with a monoclonal antibody against BDCA-1 and envisioned with a secondary goat anti-mouse antibody conjugated with AF568 (red, column 2). Column 3 is the result of merging columns 1 and 2. Representative microphotographs (×100) of cDCs are shown 24 h after the first challenge at the diluent (a) and allergen sites (b) and after the sixth challenge at the allergen site (c). Tissue cDCs significantly increased 24 h after the first allergen challenge compared to the diluent, and their numbers remained high after the sixth allergen challenge, although the difference was not significant (d). A significant inverse correlation was found between blood and skin tissue-infiltrating cDCs 24 h after the first allergen challenge (*P* = 0.0202; *r* = −0.667) (e). Data are expressed as median with interquartile range (first and third quartiles). *P < 0.05, WBC: whole blood cells.

DC subsets were examined in the skin at the allergen and diluent sites 24 h after the first and sixth challenges, time-points at which DC infiltration has been reported to peak [29]. Our findings demonstrated that pDCs and cDCs were mostly located in the subepithelium within 150 *μ*m range from the epidermis but occasionally also extended deeper into the dermis (Figures 4(a)–4(c) and 5(a)–5(c), resp.). Pertinent to pDC recruitment, there was a trend for increased pDC numbers at the allergen site after the final allergen challenge (Figure 4(d)). In contrast, one-way ANOVA revealed significant differences between the distinct time-points for cDCs (*P* = 0.0015), with* post hoc* analysis demonstrating a significant increase at the allergen site 24 h after the first allergen skin challenge, compared to the diluent site (*P* < 0.05, Figure 5(d)). Although a trend for increased cDCs was also observed at the allergen site 24 h after the sixth challenge compared to the diluent site, this difference was not significant according to* post hoc* analysis (Figure 5(d)). Importantly, we observed that the percentages of circulating cDCs inversely correlated with those of skin-infiltrating cDCs (*P* = 0.0202; *r* = −0.6667, Figure 5(e)). No correlations were observed between DC subset numbers at the skin site and the magnitude of the early- or late-phase skin reactions (data not shown). In summary, these data provide evidence that cDCs are summoned to the inflamed skin site early following* in vivo* allergen exposure.

### 3.4. Activin-A Is Increased in the Skin after Repeated* In Vivo* Allergen Challenges and Correlates with cDC Numbers

**Figure 6:**
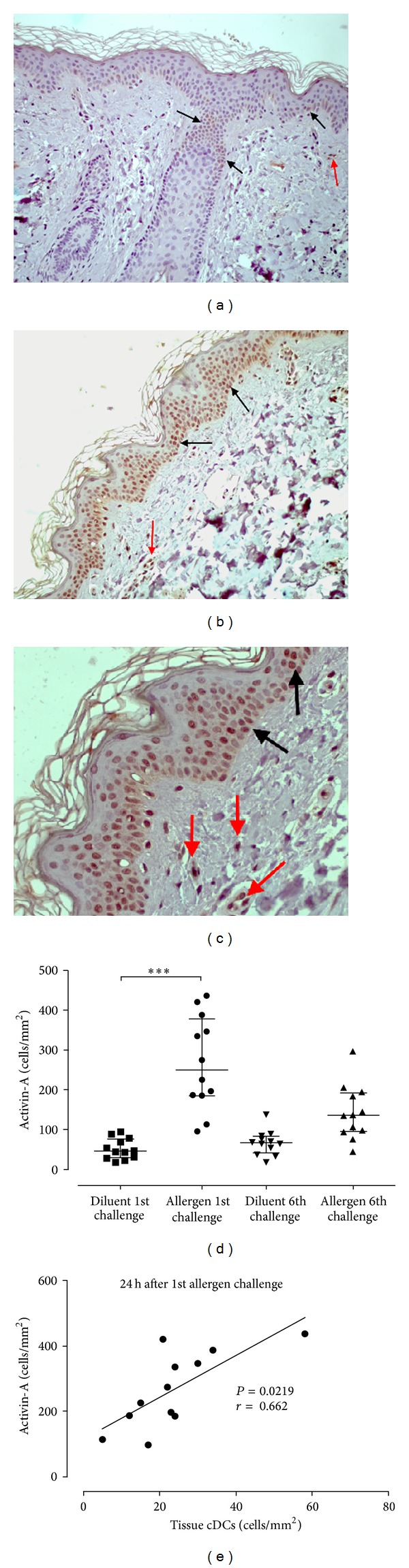
Activin-A is increased in the skin after allergen challenge and correlates with BDCA^+^ cDCs. Activin-A in the skin was analysed using the alkaline phosphatase-antialkaline phosphatase method (red colour). (a) Activin A in normal skin (diluent site) was minimal and mostly located in a scattered fashion in the basal cells of the epidermis (black arrows) with minimal expression in the dermis (red arrows) (×100). (b) After the first allergen challenge, activin-A was more prominent and intense in the epidermis (black arrows) and in infiltrating inflammatory cells (red arrows) in the dermis (×100), also shown in (c) at higher magnification (×200). (d) Quantification of activin-A^+^ cells showed significantly higher expression at the allergen site 24 h after the first challenge compared to the diluent site. (e) The numbers of activin-A^+^ cells correlated with the numbers of BDCA-1^+^ cDCs in the dermis 24 h after allergen challenge (*P* = 0.0219; *r* = 0.662). Data are expressed as median with interquartile range (first and third quartiles). *P < 0.001.

Our data showed that activin-A expression in normal skin (diluent site) was minimal and mostly present in a scattered fashion at the basal cells of the epidermis with very low levels observed in the dermis (Figure 6(a)). Interestingly, after the first* in vivo* allergen skin challenge, activin-A expression was more prominent and intense in the epidermis, as well as, in infiltrating inflammatory cells in the dermis (Figures 6(b) and 6(c)). Activin-A was also observed at low levels in the connective tissue. Following the sixth allergen challenge, activin-A in the epidermis and dermis at the allergen site was higher compared to the diluent site, but to a lesser extent, and intensity compared to that after the first allergen challenge (Figure 6(d)). As cDCs showed a similar kinetic pattern to that of activin-A following* in vivo* allergen challenge, we investigated the existence of possible correlations. Interestingly, the number of activin-A^+^ cells correlated with the number of BDCA-1^+^ cDCs at the 24 h time-point after allergen challenge (*P* = 0.0219; *r* = 0.662, Figure 6(e)). Together, these findings suggest a role for activin-A at the inflamed skin site during* in vivo* allergen challenge.

### 3.5. Activin-A Modulates the Chemokine Receptor Profile of Allergen-Stimulated CD1c^+^ DCs

**Figure 7:**
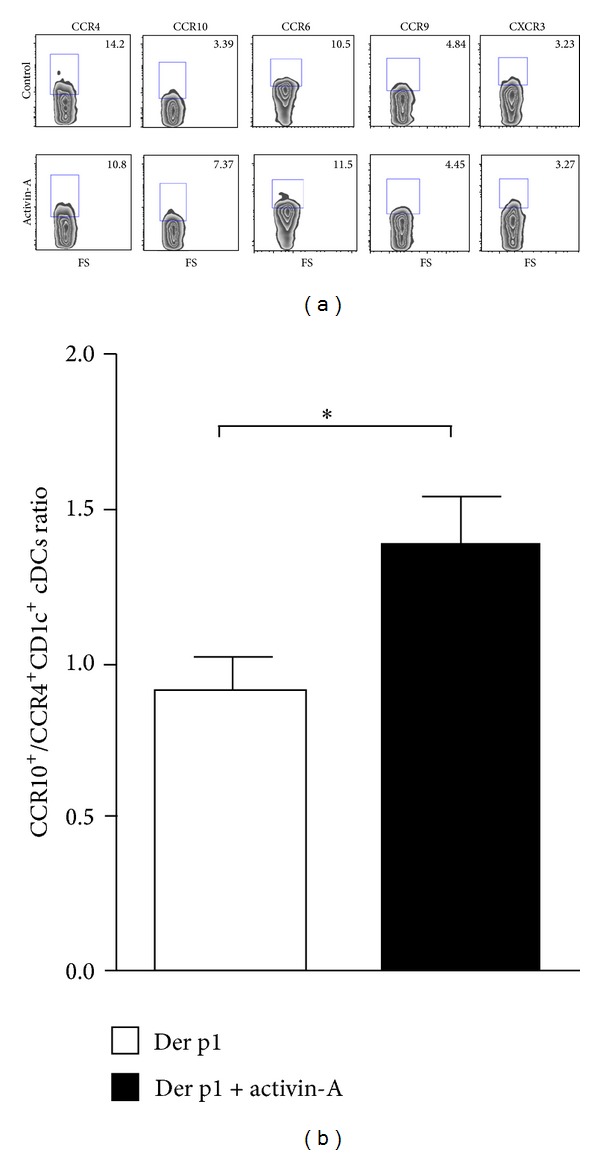
Activin-A modifies the chemokine receptor profile of CD1c^+^ cDCs towards a skin-homing phenotype. CD1c^+^ cDCs were isolated from the peripheral blood of individuals with atopy to* Dermatophagoides pteronyssinus* (Der p1), cultured for 24 h with 1 *μ*g/mL Der p1 in the presence of 50 ng/mL recombinant activin-A or PBS (control). DCs were stained with fluorochrome-conjugated antibodies against human CCR4, CCR10, CCR6, CCR9, and CXCR3 and analysed by flow-cytometry. (a) Activin-A induced an increase in CCR10 levels, concomitant with a decrease in CCR4 on CD1c^+^ cDCs during stimulation with Der p1* in vitro*. No differences were observed regarding the expression of CCR6, CCR9, and CXCR3 in Der p1-stimulated CD1c^+^ cDCs in the presence or absence of activin-A. (b) Activin-A significantly increased the ratio of CCR10/CCR4 expressing cDCs. Data are representative of two independent experiments. *P < 0.05.

Previous studies have demonstrated that human cDCs express higher levels of activin-A type I and II signalling receptors compared to pDCs and respond actively to the ligand [24]. In view of our findings showing a strong correlation between activin-A expression and cDC trafficking at the inflamed skin site upon allergen encounter, we hypothesized that activin-A may affect the cDC chemotactic profile. To address this, we examined the effects of activin-A on chemokine receptor expression by cDCs isolated from the peripheral blood of atopics during stimulation with allergen* in vitro*. No significant differences were observed in the levels of CXCR3, CCR6, or CCR9 on cDCs upon activin-A treatment (Figure 7(a)). Activin-A, however, increased the expression of CCR10 on CD1c^+^ DCs, concomitant with a decrease in CCR4, as compared to control (PBS) (Figure 7(a)). In fact, activin-A significantly increased the ratio of CCR10/CCR4 expressing cDCs (Figure 7(b)). Chemokine receptor levels were very low in nonstimulated cDCs (medium alone) and increased upon LPS treatment (data not shown). Overall, these data suggest that activin-A may be involved in retaining cDCs in the skin after allergen challenge through the regulation of CCR4 and CCR10 expression.

## 4. Discussion

Human DC kinetics have been examined in the blood [12, 13], induced sputum [14], or bronchial tissue [15] of asthmatics, following a single allergen inhalation or segmental allergen challenge. Still, simultaneous evaluation of DC kinetics at the periphery and the challenged tissue site has not been investigated. A single study has examined DC kinetics in the blood and bronchoalveolar lavage fluid (BALF) in seven asthmatics after a single segmental allergen challenge but did not evaluate DC numbers at the inflamed bronchial tissue [16]. Jahnsen et al. investigated DC kinetics in the periphery and the challenged nasal tissue, but the study was limited to pDCs [17]. To our knowledge, this is the first human study evaluating both cDC and pDC kinetics concomitantly in the periphery and at the inflamed tissue site after repeated* in vivo* allergen skin challenges in atopic subjects, encapsulating both the initial and chronic stages of allergic inflammation.

Experimental skin allergen challenge has been widely used to examine the cellular processes associated with early- and late-phase allergic reactions [30] and to study the effects of repeated allergen exposure in the airways [26]. Moreover, these models imitate the chronicity of allergen exposure in a more standardized fashion and, as late-phase skin reactions exhibit similar histopathological inflammatory patterns to that of the airways [26], they can be used to recapitulate chronic allergic airway inflammation [31]. Furthermore, these models demand only atopic status as a prerequisite, which usually requires no treatment. In asthma, however, treatment with corticosteroids is often warranted, which can act as a confounding factor by decreasing pDC absolute numbers and cytokine responses upon* in vivo* bronchial allergen challenge [32]. Moreover, steroid treatment has been recently shown to increase the capacity of GILZ^+^ circulating DCs to activate allergen-specific IL-10 Tregs in allergic patients [33]. In addition, the difficulties and increased patient risk associated with performing multiple bronchial allergen challenges in conjunction with consecutive endobronchial biopsies represent critical restraining factors. Still, our study presents certain limitations, mainly pertaining to the fact that we did not perform challenges with an irrelevant allergen nor did we include healthy nonatopic controls to confirm that our findings were due to allergen exposure* per se*.

There has been contradicting evidence pertinent to the effects of repeated allergen challenges in the nose, airways, and skin of atopics. In certain studies, multiple challenges led to tolerance while in others led to enhanced inflammatory cell migration [30]. The time interval between skin challenges, the type of allergen used, and the dosing scheme is crucial in determining priming versus tolerance. Intervals longer than than one week seem to induce small late-phase reactions [26], while challenges with house dust mite often result in an increase in late skin reactions compared to grass pollen [30]. We observed early- and late-phase skin reactions after every challenge with house dust mite and detected no differences between challenges, as also shown by other investigators [31]. These data suggest that the possibility of desensitization is minimal.

Plasmacytoid DCs are primarily observed in peripheral blood, but they also migrate to lymphoid organs or tissues during inflammation [34]. The finding that there were no differences in the numbers of skin pDCs after repeated allergen challenges was rather unexpected. Previous studies of bronchial allergen challenge in asthmatics have also failed to show an accumulation of pDCs in the bronchial tissue [15]. One could argue that single allergen challenge models could be insufficient for the recruitment of pDCs; still, we did not observe significant changes in pDCs even after six weekly allergen challenges, although a trend was detected. The observation that pDC levels were decreased in the periphery within one hour after allergen challenge is suggestive of a rapid recruitment of these cells to the exposed skin site, possibly occurring long before 24 h. Moreover, pDCs have been shown to respond selectively to homeostatic chemokines, demonstrating an inherent tendency to migrate to secondary lymphoid organs rather than to inflammatory tissue sites [35]. It is, therefore, possible that peripheral pDCs were recruited predominantly to local draining lymph nodes (DLNs).

Conventional DCs, in contrast to pDCs, circulate in the blood or reside in peripheral tissues [34] and are quickly depleted from the circulation following allergen inhalation in asthmatics [12]. Upon local allergen challenge, cDCs have been reported to accumulate within the bronchial mucosa [15]. It is, however, not clear whether this is due to an enhanced cDC migration from the periphery, the differentiation of DCs* in situ*, or decreased migration of tissue-resident DCs towards DLNs upon allergen encounter [36]. Our findings demonstrate that cDCs accumulate at the inflamed skin site within 24 h after local allergen challenge and inversely correlate with their numbers in the periphery at the same time-point. These data support the notion that cDCs are recruited from the bloodstream to the skin early following allergen exposure. Still, this does not preclude the possibility of local DC differentiation from precursor populations. In fact, increased numbers of CD34^+^ progenitor cells have been noted in the airways of asthmatics [37]. In accordance with our findings, cDCs have also been reported to decrease in the periphery and increase in BALF 24 h after segmental allergen challenge in mild asthmatics; however, possible correlations were not examined [16]. Corrigan et al. also reported increased numbers of C11c^+^ BDCA-1^+^ cDCs bearing the thymic stromal lymphopoietin receptor in the skin, 24 h after allergen challenge, and their data supported that these cells were recruited from the circulation rather being differentiated* de novo* [29].

In order to investigate tissue mDCs, we used BDCA-1 as a cellular marker in the skin. CD1a has also been used to identify cDCs in normal skin; however, CD1c (BDCA-1) is considered a more useful marker as it colocalizes with nearly all CD11c+ cells [4, 5]. Upon inflammation, an additional population of infiltrating inflammatory DCs has been reported that is different from steady state dermal cDCs and is considered their potential precursor [4, 38]. In the acute phase of atopic dermatitis, these cells coexpress CD1b/c and produce chemokines that attract T_H2_ cells [4, 38, 39]. It is unknown whether the cDCs examined in the present studies contain also a population of these highly inflammatory DCs. Delineation of the precise phenotypic and functional properties of cDCs recruited to the skin upon* in vivo* allergen challenge will help resolve this question.

Our results also demonstrate that activin-A is greatly increased in the skin after* in vivo* allergen challenge. Importantly, its expression correlates with the numbers of infiltrating cDCs at 24 h after challenge, suggesting that activin-A may be involved in cDC recruitment to the skin. Activin-A induces DC migration and can act as a differentiating factor for blood cDCs and lung Langerhans cell-like DCs [24, 25]. Although cDCs express both type I and II receptors [40], activin-A has not been shown to exert direct chemotactic effects on these cells. Still, activin-A alters the chemokine and the chemokine receptor profile of human Langerhans dendritic cells [24, 25]. In agreement, we demonstrate that activin-A induced a reciprocal shift up on CCR10, with a shift down on CCR4 on allergen-stimulated CD1c^+^ cDCs obtained from atopic subjects. Both receptors are involved in DC and T cell trafficking to the skin and inflamed epithelia, including the lungs [41–43]. An inversion of the fold increase ratio in CCR4/CCR10 expression in skin allografts has been previously associated with T cell trafficking and allograft rejection [44]. Moreover, a recent single study has shown that circulating pDCs express increased levels of CCR4 and CCR10 in patients with allergic asthma [32]. Together, these findings prompt the speculation that activin-A may be involved in retaining cDCs at the inflamed skin site, at least partly, through modulating CCR10/CCR4 expression.

In summary, our study highlights key differences in the trafficking of human pDCs and cDCs upon* in vivo* allergen challenge in the skin, pointing to distinct roles in the allergic response. Importantly, our findings facilitate the understanding of human DC behaviour during allergen encounter* in vivo*, which is essential for controlling the balance between immunity and tolerance in allergic diseases.

## Figures and Tables

**Table 1:** Subject characteristics and summary of results for peripheral and tissue DC subset recruitment.

	Subject 1		Subject 2		Subject 3		Subject 4		Subject 5		Subject 6		Subject 7		Subject 8		Subject 9		Subject 10		Subject 11		Subject 12	
Gender	M		F		F		M		F		F		M		M		F		F		M		F	
Age	30		53		23		30		31		28		40		31		31		27		26		28	
IgE	830		1100		720		54		106		71		19		19		44		235		630		120	

	Peripheral dendritic cells (% of total WBC)																							
cDCs baseline	0,3986		0,209		0,2445		0,1706		0,2122		0,2147		0,1283		0,2223		0,2102		0,1334		0,178		0,175	
cDCs 1 h	0,3602		0,2454		0,2016		0,1544		0,1804		0,1845		0,1421		0,1887		0,1778		0,174		0,154		0,1444	
cDCs 24 h	0,3731		0,1571		0,2424		0,1089		0,2424		0,1908		0,1296		0,1777		0,1132		0,1278		0,1057		0,1011	
cDCs 5 w	0,2706		0,2307		0,2923		0,1262		0,174		0,2711		0,1527		0,1368		0,1593		0,1124		0,1162		0,1282	
pDCs baseline	0,1284		0,1071		0,2468		0,1213		0,225		0,2554		0,1139		0,1798		0,0553		0,1413		0,2369		0,244	
pDCs 1 h	0,1102		0,105		0,1895		0,0828		0,1708		0,1681		0,1175		0,1105		0,0581		0,0524		0,167		0,104	
pDCs 24 h	0,0859		0,1313		0,2099		0,0732		0,144		0,1982		0,119		0,1503		0,0647		0,1526		0,212		0,2059	
pDCs 5 w	0,0214		0,0013		0,2119		0,066		0,3738		0,1559		0,1715		0,078		0,1308		0,0907		0,2113		0,198	

	Tissue dendritic cells (cells per mm^2^)																							
	ALL	DIL	ALL	DIL	ALL	DIL	ALL	DIL	ALL	DIL	ALL	DIL	ALL	DIL	ALL	DIL	ALL	DIL	ALL	DIL	ALL	DIL	ALL	DIL

cDCs 24 h	5	3	24	8	15	7	34	12	12	5	21	13	58	25	24	19	17	5	22	5	23	9	30	8
cDCs 5 w	14	6	16	8	20	13	10	13	23	10	30	8	25	15	15	48	29	17	25	5	27	6	14	6
pDCs 24 h	12	10	6	8	8	5	12	15	4	4	24	14	5	2	5	3	3	2	18	10	7	3	12	10
pDCs 5 w	10	8	12	5	4	25	8	6	9	6	11	5	14	10	10	16	6	3	4	1	5	4	5	8

## Abbreviations

AD: Atopic dermatitis
BALF: Bronchoalveolar lavage fluid
BDCA: Blood dendritic cell antigen
CCR: C-C motif chemokine receptor
cDC: Conventional dendritic cell
CXCR: CXC motif chemokine receptor
DC: Dendritic cell
Der p1: * Dermatophagoides pteronyssinus*
DLN: Draining lymph node
EPR: Early-phase reaction
HDM: House dust mite
IF: Immunofluorescence
IHC: Immunohistochemistry
LC: Langerhans cell
LPR: Late-phase reaction
LPS: Lipopolysaccharide
pDC: Plasmacytoid dendritic cell
SPT: Skin prick test.
